# Assembly of Abundant and Rare Bacterial and Fungal Communities in Different Typical Forest Types in the Zhongtiao Mountains

**DOI:** 10.3390/microorganisms13081911

**Published:** 2025-08-16

**Authors:** Zixing Li, Ran Wang, Mengtao Zhang

**Affiliations:** College of Forestry, Shanxi Agriculture University, Jinzhong 030801, China; lizixing0927@163.com (Z.L.); 18536494038@163.com (R.W.)

**Keywords:** abundant microbial communities, rare microbial communities, soil bacteria, soil fungi, pure forests, mixed forests

## Abstract

Soil microorganisms play an important role in maintaining the functioning of terrestrial ecosystems. Soil microbial communities usually contain both abundant and rare microorganisms. However, in forest ecosystems, the differences in the functions and assembly processes of abundant and rare microbial taxa in soils between planted pure and mixed forests are currently unknown. In this study, four different forest types in the Zhongtiao Mountains were selected, and the diversity and assembly processes of abundant and rare microbial communities in their soils were quantitatively analyzed. The results show that there are differences in the diversity and assembly processes of abundant and rare microorganisms in the four forests. Significant differences in the α-diversity (Shannon index) of abundant bacteria (*p* = 0.019) and rare fungi (*p* = 0.049) were obtained in the four forests. The assembly of abundant bacterial and fungal communities in the four forest types was mainly influenced by stochastic processes, the assembly of rare bacterial communities was mainly influenced by deterministic processes, and the assembly of rare fungal communities was influenced by a combination of deterministic and stochastic processes. Planted mixed forests increase the relative contribution of deterministic processes in the assembly of rare fungal communities compared to planted pure forests. This study determined the relative contributions of deterministic and stochastic processes in the assembly of abundant and rare microbial communities among different forest types, providing a theoretical basis for forest management in mixed forests.

## 1. Introduction

Soil is an important component of terrestrial ecosystems, and as the most diverse group of organisms in soil and the main decomposers of soil organic matter, soil microorganisms are involved in biogeochemical cycles [1,2] and play an important role in maintaining terrestrial ecosystem functions [3]. Soil microorganisms are also essential in carbon sequestration and decomposition [4,5,6], soil bacteria are capable of metabolizing a wide range of compounds [7], and soil fungi are key decomposers in soil [8]. The abundance of species in soil bacterial and fungal communities is usually unevenly distributed [9], including a small number of abundant species and a large number of rare species, and abundant and rare microbial communities are important components of soil microbial communities. Abundant and rare microbial communities usually exhibit different functional properties, and their distribution patterns differ [10,11]. Overall, both abundant and rare microorganisms are able to maintain ecosystem stability [12]. Abundant microbes are more adaptable to their environment, are related to the cycling of nutrients in the soil [13,14], and are more dominant in the competition for carbon resources [15]. Rare microorganisms act as a reservoir of diversity that enhances bacterial resilience and resistance in disturbed environments [16]. A study showed that rare microorganisms were able to maintain the multifunctionality of ecosystems in long-term fertilized soils [17]. Therefore, the study of the community composition of abundant and rare microorganisms in soil is of great interest to understand the function of soil microorganisms in ecosystems.

As a recognized microbial reservoir in terrestrial ecosystems [18], soils make an important contribution to microbial diversity and dispersal [19]. Microbial community assembly can be affected by different ecological processes [11], which are usually classified as deterministic and stochastic. Deterministic processes are environmental selection induced by habitat conditions and competition, antagonism, and exclusion between different species [20]. Stochastic processes are random birth/death events, probabilistic diffusion, and unpredictable perturbations [21]. Quantifying the relative contributions of deterministic and stochastic processes in microbial communities is essential for the conservation of soil ecosystem function [22,23]. Past studies have shown that microbial assembly processes are linked to environmental changes [24,25]. Abundant microbes in the ocean are subject to more dispersal limitations than rare microbial communities [10]. In contrast, rare microbial communities were more limited than abundant microbial communities in lakes [26], agricultural ecosystems [11], and grasslands on the Tibetan Plateau [27]. For planted forests, a study on the effects of different management and planting practices on soil microbial communities in *Eucalyptus* plantations showed that the aggregation of abundant taxa in *Eucalyptus* plantations was dominated by stochastic processes, whereas the aggregation of rare taxa was dominated by deterministic processes [28]. A study on subtropical forest soils suggests that homogenous ecological processes play an important role in microbial community assembly at the local scale [29]. Another study on soil microbial communities in subtropical forests in China showed that key microbial communities contributed to the stability of soil microbial communities in forests to a greater extent than rare microbial communities [30]. Although these studies have elucidated the important roles of abundant and rare microbial communities in various ecosystems, the differences in the functions and assembly processes of abundant and rare microbial taxa between planted pure and mixed forests in forest ecosystems have not yet been clearly elucidated. To determine the relative contribution of abundant and rare microbial taxa to forest ecosystem processes, we hypothesized: (i) soil microbiological differences resulting from differences in canopy species in forests are mainly in rare microorganisms; and (ii) the relative contributions of deterministic and stochastic processes differ in pure and mixed forests.

## 2. Materials and Methods

### 2.1. Study Area

The study area is located in the Zhongtiao Mountains, Zhongcun Forestry, Qingshui County, Jincheng City, Shanxi Province, China (35°24′00″~35°40′00″ N, 111°56′12″~112°14′00″ E). The area is located in the south of Shanxi Province, in the middle of Zhongtiao Mountain forest area, belonging to the warm temperate semi-humid continental monsoon climate zone. The altitude is 1200–1700 m, the average annual temperature is 10.3 °C, the annual sunshine hours are 2679.8 h, the frost-free period is 197 d, the average annual precipitation is about 700–900 mm, and the precipitation is mainly concentrated in the summer (June–September). The rocks in the area are mostly limestone. According to Genetic Soil Classification of China, the soil types in the region are mainly meadow soil, brown forest soil, drench brown soil, and brown soil. The main trees planted in the region are *Pinus tabuliformis*, *Pinus armandi*, *Quercus mongolica*, *Quercus variabilis*, *Populus davidiana*, and *Betula platyphylla*.

**Figure 1:**
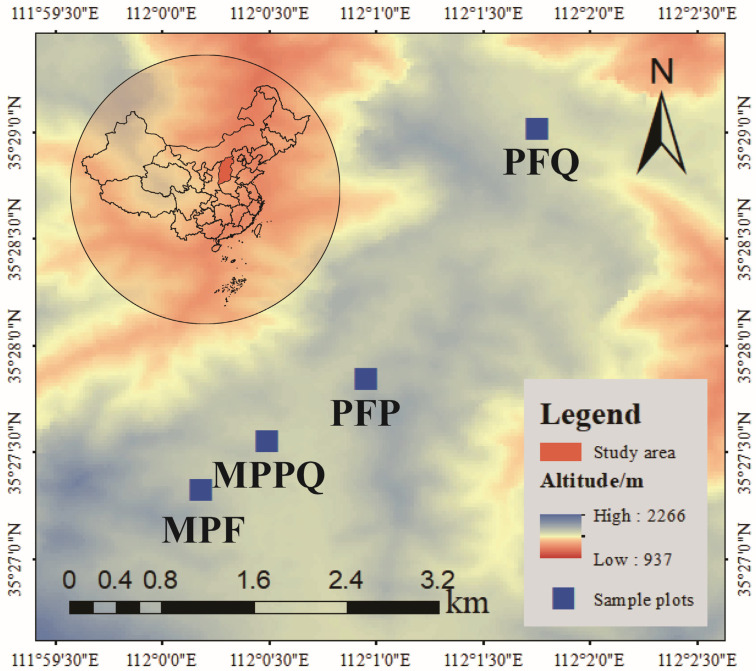
Location of the study area.

In July 2024, sample plots of four different forest types were set up in an area with similar standing conditions in the XiaChuan Management Area of the Zhongcun Forest (Figure 1):

(i) Mixed Planted Forest (MPF): The main tree species in the sample plot were *Pinus tabuliformis*, *Quercus mongolica*, *Populus davidiana*, *Betula platyphylla*, and *Carpinus turczaninovii*. A total of 32 trees were investigated in the plot. The average tree height in the sample plot was 13.80 ± 0.49 m, and the average diameter at breast height (DBH) in the sample plot was 12.45 ± 0.68 cm;
(ii) Planted Forest of *Pinus tabuliformis* (PFP): The main tree species in the sample plot was *Pinus tabuliformis*. A total of 28 trees were investigated in the plot. The average tree height in the sample plot was 16.61 ± 4.62 m, and the average diameter at breast height (DBH) in the sample plot was 23.69 ± 3.11 cm;
(iii) Mixed Planted Forest of *Pinus tabuliformis* and *Quercus mongolica* (MPPQ): The main tree species in the sample plot were *Pinus tabuliformis* and *Quercus mongolica*. A total of 44 trees were investigated in the plot. The average tree height in the sample plot was 15.17 ± 1.32 m, and the average diameter at breast height (DBH) in the sample plot was 21.94 ± 1.61 cm;
(iv) Planted Forest of *Quercus mongolica* (PFQ): The main tree species in the sample plot was *Quercus mongolica*. A total of 44 trees were investigated in the plot. The average tree height in the sample plot was 10.87 ± 0.83 m, and the average diameter at breast height (DBH) in the sample plot was 16.03 ± 0.62 cm.

There were four replicates of each forest type, and each replicate plot was 20 m × 20 m in size, with an interval of 50 m between each two plots. The latitude, longitude, and elevation of each plot were recorded, and the species name, diameter at breast height (DBH), and height of all trees with DBH ≥ 5 in the plot were recorded.

### 2.2. Soil Sample Collection and Determination

In each 20 m × 20 m sample plot, five small sample squares of 5 m × 5 m were set up according to the five-point sampling method, and soil sampling was carried out in the center of each 5 m × 5 m small sample square. Three bags of 0–20 cm topsoil were taken at each sampling point. After sampling, the 15 bags of soil samples from each sample plot were mixed well in the laboratory, large stones and roots were removed with tweezers, and then the combined soil samples were sieved through a 0.9 mm sieve.

Total DNA from soil microorganisms was extracted using the OMEGA Soil DNA Kit (M5635–02) (Omega Bio-Tek, Norcross, GA, USA). The bacterial 16S rRNA V3–V4 region was amplified with primers 338F and 806R [31], while fungal ITS regions were amplified with ITS5 and ITS2 primers [32]. After completion of the PCR reaction, the cut target fragments were recovered using a sorted magnetic bead recovery method. PCR products were quantified using the Quant-iT PicoGreen dsDNA Assay Kit, followed by 2 *×* 250 bp bipartite sequencing using the NovaSeq 6000 SP Reagent Kit (500 cycles). Preliminary screening of the raw sequencing reads was performed based on sequence qualit; samples failing quality control were retested or supplemented.following initial control, the raw sequences were divided into libraries and samples according to their index and barcode information, and the barcode sequence were removed. Serial denoising or OTU clustering was performed according to the analysis process of QIIME2 (2019.4) dada2 or the analysis process of Vsearch software (v2.13.4 linux x86 64). Taxonomy was assigned to OTUs using the classify-sklearn naïve Bayes taxonomy classifier in feature classifier plugin against the SILVA Release 138 (Bacteria) [33] and UNITE Release 9.0 (Fungal) Database [34]. Based on the distribution of OTUs across samples, the α-diversity level (Shannon index) of each sample was evaluated, and the adequacy of sequencing depth was appropriate is reflected in the dilution curve.

### 2.3. Data Analysis

Statistical analyses were performed using Rv4.4.1. OTUs were classified as abundant or rare microorganisms based on their relative abundance. Abundant OTUs were defined as OTUs with a relative abundance > 0.1%, and rare OTUs were defined as OTUs with a relative abundance < 0.01% [35]. The α-diversity (Shannon) of soil microorganisms in the four forest types was calculated and analyzed by one-way ANOVA and least significant difference (LSD) multiple comparisons to determine whether there were significant differences in the α-diversity (Shannon) of soil microorganisms among the different forest types. The structure of soil microbial communities (abundant bacteria, rare bacteria, abundant fungi, and rare fungi) was analyzed using the Bray–Curtis similarity matrix and principal coordinate analysis (PCoA). Differences in soil microbial β-diversity among the four forest types were determined using permutation multivariate analysis of variance (PERMANOVA) with Adonis function [36]. The homogeneity of the dispersion was verified using the beta-dispersion test. The phylogenetic composition of soil microbial communities in four different forest types was determined by calculating the nearest taxon index (NTI). For a single community, a mean NTI > 0 indicates that coexisting taxa are more phylogenetically clustered, and a mean NTI < 0 indicates that coexisting taxa are more phylogenetically over-dispersed [35]. One-way ANOVA and least significant difference (LSD) multiple comparisons were used to analyze whether there were significant differences in NTI between forest types. The assembly processes of soil microbial communities were determined by calculating the β nearest taxon index (βNTI) and Bray–Curtis-based Raup–Crick (RCbary) values [37]. When |βNTI| > 2.0, the key assembly process for community composition was deterministic (homogeneous selection, βNTI < −2.0; heterogeneous selection, βNTI > 2.0); when |βNTI| < 2.0, the key assembly process for community composition was stochastic [38]. When |RCbary| > 0.95, communities are considered as driven by dispersal (homogenizing dispersal, RCbary < −0.95; dispersal limitation, RCbary > 0.95). When |βNTI| < 2 and |RCbary| < 0.95, the communities were driven by undominated processes [39].

## 3. Results

### 3.1. Composition of Abundant and Rare Microorganisms in Different Forest Types

**Figure 2:**
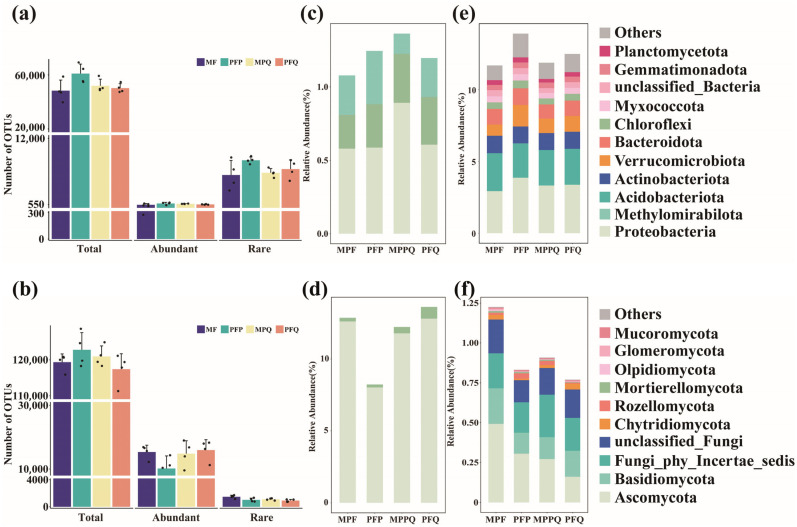
Distribution of microorganisms in soils of four forest types. (**a**) Number of OTUs for total, abundant, and rare bacteria. (**b**) Number of OTUs for total, abundant, and rare fungi. (**c**) Relative abundance of abundant bacterial dominant communities in phylum classifications. (**d**) Relative abundance of abundant fungal dominant communities in phylum classification. (**e**) Relative abundance of rare bacterial dominant communities in phylum classification. (**f**) Relative abundance of rare fungal dominant communities in phylum classifications.

After smoothing according to the minimum sample sequence, an average of 95,567 bacteria feature sequences and 128,003 fungi feature sequences were obtained for each sample for further analysis (Figure S1 and Table S1). Among bacteria, an average of 1.19% of the bacterial OTUs were identified as abundant bacteria for each sample, and an average of 12.50% of the bacterial OTUs were identified as rare bacteria for each sample (Figure 2a and Table S2). Among the fungi, an average of 11.70% of the fungi OTUs were identified as abundant fungi for each sample, and an average of 0.93% of the fungi OTUs were identified as rare fungi for each sample (Figure 2b and Table S2).

Proteobacteria, Chloroflexi, and Methylomirabilota comprised the abundant bacterial taxa (Figure 2c). Proteobacteria, Acidobacteriota, and Actinobacteriota were the dominant rare bacterial taxa, with relative abundance accounting for more than 5% of the total rare bacterial sequences (Figure 2e). MPPQ had the highest total relative abundance of abundant bacteria among the four forest types, followed by PFQ, which had the highest total relative abundance of rare bacteria among the four forest types. Ascomycota and Mortierellomycota together formed the abundant fungal taxa (Figure 2d). Ascomycota, Basidiomycota, and Chytridiomycota were the dominant rare fungal taxa (Figure 2f). Among them, PFP had the lowest total relative abundance of abundant and rare fungal taxa among the four forest types.

**Figure 3:**
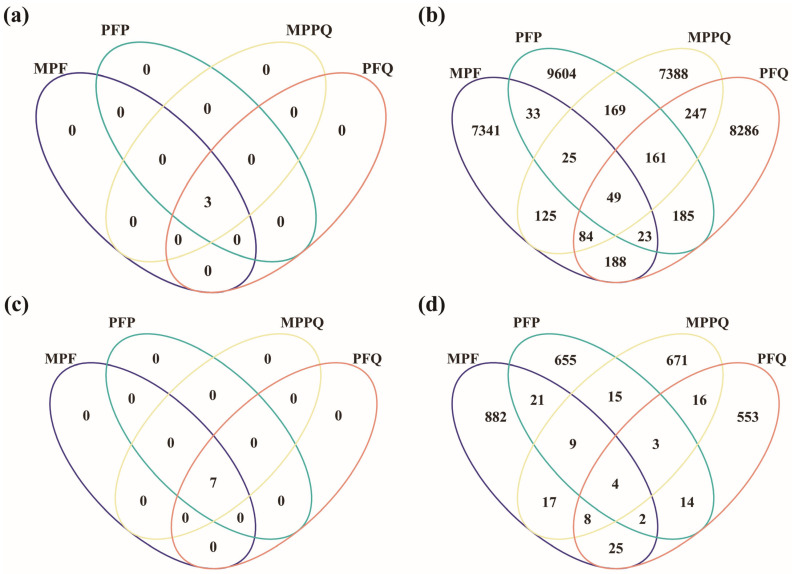
Soil microbial community composition in different forest types. (**a**) Number of common and unique abundant bacteria species. (**b**) Number of common and unique rare bacteria species. (**c**) Number of common and unique abundant fungi species. (**d**) Number of common and unique rare fungi species.

Between the different forest types, abundant bacteria (Figure 3a) and fungi (Figure 3c) showed that all species were shared, and rare bacteria (Figure 3b) and fungi (Figure 3d) showed that the number of unique species was greater than the number of shared species. The number of unique species of rare bacteria in MPF accounted for 21.6% of the total number of rare bacteria, 28.3% of the total number of rare bacteria in PFP, 21.8% of the total number of rare bacteria in MPPQ, and 24.4% of the total number of rare bacteria in PFQ. The number of unique species of rare fungi accounted for 30.4% of the total number of rare fungi in MPF, 22.6% of the total number of rare fungi in PFP, 23.2% of the total number of rare fungi in MPPQ, and 19.1% of the total number of rare fungi in PFQ.

### 3.2. Diversity of Abundant and Rare Microbial Communities in Different Forest Types

**Figure 4:**
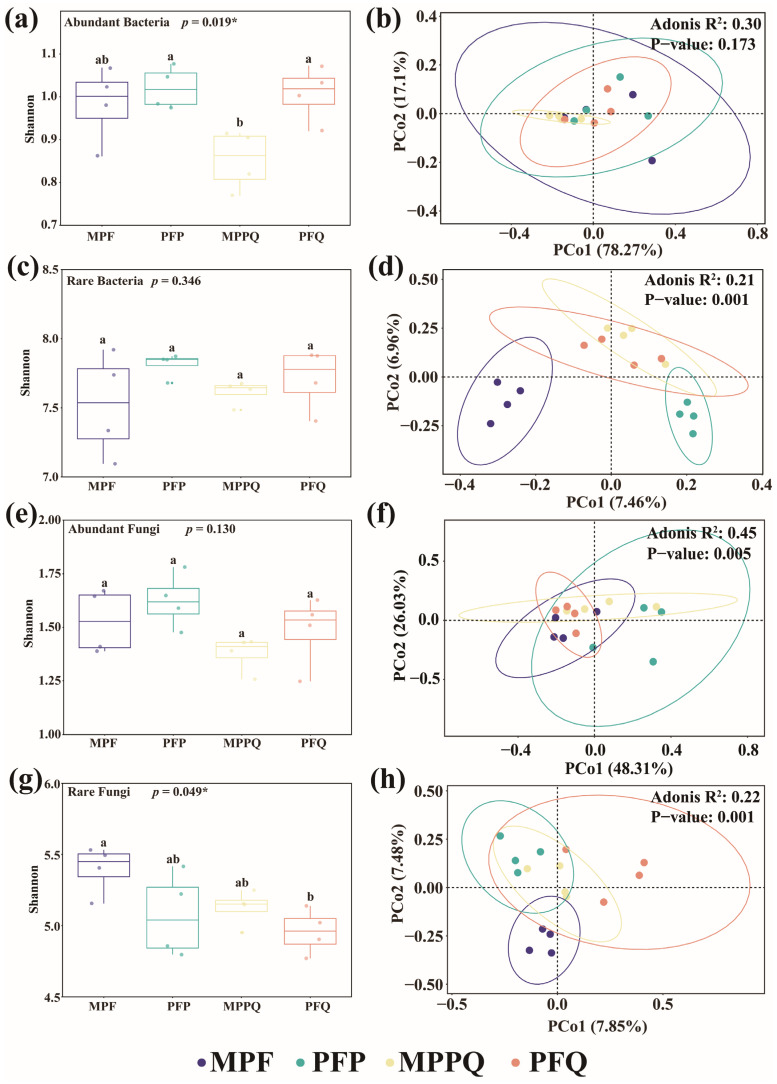
Diversity of soil microorganisms in different forest types. Shannon index of (**a**) abundant bacteria, (**c**) rare bacteria, (**e**) abundant fungi, and (**g**) rare fungi. Differential patterns of β-diversity of (**b**) abundant bacteria, (**d**) rare bacteria, (**f**) abundant fungi, and (**h**) rare fungi. Different letters indicate that Shannon index was significantly different (*p* < 0.05) among the four forest types. Asterisks denote significance levels (* *p* < 0.05).

The rare bacteria (Figure 4c) and fungi (Figure 4g) communities had higher Shannon index values than the abundant bacteria (Figure 4a) and fungi (Figure 4e) communities. The Shannon index of abundant bacteria was significantly different among the four forest types, with the Shannon index values of MPPQ being significantly lower than those of PFP and PFQ, while the Shannon index value of rare fungi was significantly different among the four forest types, with the Shannon index values of PFQ being significantly lower than those of MPF (Table S3).

The β-diversity of soil microbial communities was analyzed at the OTU level using principal coordinate analysis (PCoA) based on the Bray–Curtis distance matrix. The results showed that the community composition of soil abundant bacteria (Figure 4b) was not significantly different among forest types, and the community composition of soil rare bacteria (Figure 4d) was significantly different among forest types. The community composition of soil abundant fungi (Figure 4f) and rare fungi (Figure 4h) were both significantly different among forest types. Among soil bacteria, the first two axes of abundant bacteria accounted for 95.37% of the forest type variation, and the first two axes of rare bacteria accounted for 14.42% of the forest type variation. Among soil fungi, the first two axes of abundant fungi accounted for 74.34% of the forest type variation, and the first two axes of rare fungi accounted for 15.33% of the forest type variation. There was no significant difference (F < 3.49, *p* > 0.05) in the beta-dispersion test results between abundant and rare microorganisms. The results indicate that the significance of PERMANOVA (*p* < 0.05) reflects that the true community structure differences are not caused by differences in within-group variation (Figure S2).

### 3.3. Assembly Processes of Abundant and Rare Microorganisms in Different Forest Types

**Figure 5:**
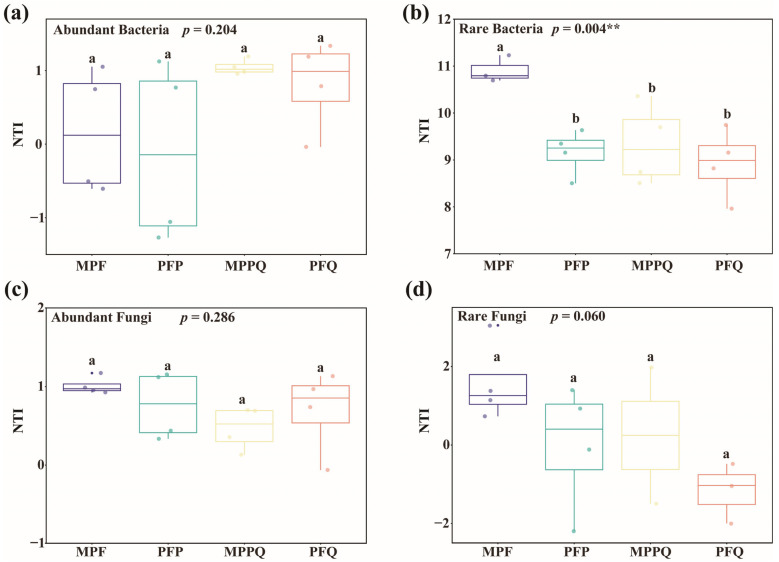
NTI of soil microorganisms in different forest types. (**a**) Abundant bacteria, (**b**) rare bacteria, (**c**) abundant fungi, and (**d**) rare fungi. Different letters indicate that NTI was significantly different (*p* < 0.05) among the four forest types. Asterisks denote significance levels (** *p* < 0.01).

In abundant bacteria (Figure 5a), the mean NTI values of MPF, MPPQ, and PFQ were all greater than zero, the mean NTI value of PFP was less than zero, and there was no significant difference in the mean NTI values among the four forest types (*p* > 0.05). In rare bacteria (Figure 5b), the mean NTI values of all four forest types were greater than zero, and the mean NTI values of the four forest types were significantly different from each other (*p* < 0.01). In abundant fungi (Figure 5c), the mean NTI values of all four forest types of soil were greater than zero, and there was no significant difference in the mean NTI values between the four forest types (*p* > 0.05). In rare fungi (Figure 5d), the mean NTI values of MPF and PFP were greater than zero, the mean NTI values of MPPQ and PFQ were less than zero, and there was no significant difference in the mean NTI values among the four forest types (*p* > 0.05) (Table S4).

**Figure 6:**
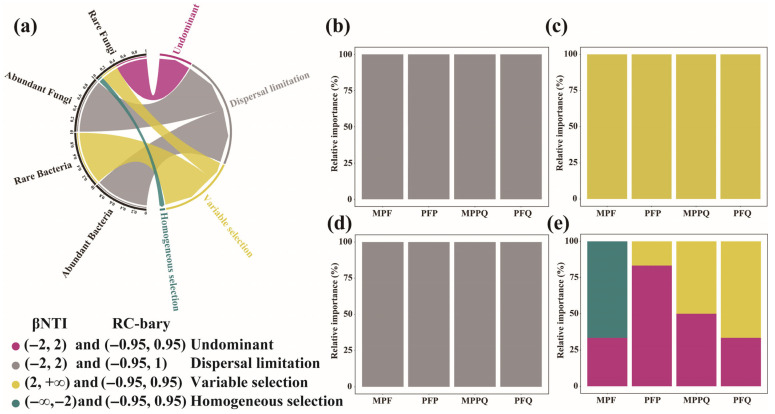
Determinism and stochasticity in community assembly. (**a**) Relative influences of deterministic and stochastic assembly processes in shaping abundant and rare bacterial and fungal communities. (**b**) Difference in determinism and stochasticity in community assembly of abundant bacteria in different forest types. (**c**) Difference in determinism and stochasticity in community assembly of rare bacterial communities in different forest types. (**d**) Difference in determinism and stochasticity in community assembly of abundant fungal communities in different forest types. (**e**) Difference in determinism and stochasticity in community assembly of rare fungal communities in different forest types.

The βNTI values of the four forest types of rich bacterial and fungal communities ranged from −2.0 to 2.0, and their assembly processes were all regulated by stochastic processes (Figure 6a), with the aggregation of the four forest types of abundant bacterial (Figure 6b) and fungal (Figure 6d) communities driven mainly by dispersal limitation. In contrast, the assembly processes of the four forest types of rare bacterial (Figure 6c) and fungal (Figure 6e) communities differed. The βNTI values of the four forest types of rare bacteria were all > 2.0, indicating that the assembly of the four forest types of rare bacterial communities was regulated by deterministic processes, and the aggregation of the four forest types of rare bacterial communities was driven by heterogeneous selection processes. The βNTI value of MPF rare fungi was <−2.0, suggesting that the assembly of rare fungal communities was regulated by deterministic processes, and the aggregation of rare fungal communities was driven by homogeneous selection processes. The βNTI value of PFP rare fungi ranged from −2.0 to 2.0, suggesting that the assembly of rare fungal communities in this forest type was regulated by stochastic processes. The βNTI values of MPPQ and PFQ rare fungi were both > 2.0, suggesting that deterministic processes regulated the assembly of rare fungal communities in these two forest types and that the aggregation of the bacterial communities in all the four forest types was driven by a process of heterogeneous selection.

## 4. Discussion

### 4.1. Differences in Abundant and Rare Microbial Community Composition and Diversity in Different Forest Types

The communities of abundant and rare microorganisms differed significantly between forest types. For bacteria, the total relative abundance of MPF abundant bacteria was the lowest among the four forest types, and the total relative abundance of rare bacteria was higher in planted mixed forests than planted pure forests. This suggests that tree species composition is an important driver of bacterial composition [40], and that differences in soil microorganism composition between planted pure and mixed forests are mainly reflected in differences in the composition of rare bacteria. For fungi, the total relative abundance of abundant fungi in PFP was the lowest among the four forest types, and PFP, with its single canopy species composition, had fewer key abundant fungi in the soil, weaker linkages within the microbial community, and less multifunctional soil ecosystems [41]. The total relative abundance of abundant fungi in PFQ was the highest among the four forest types, and the total relative abundances of abundant fungi in MPF and MPPQ were closer. This suggests that the relative abundance of abundant fungi was lower in forests planted with either coniferous species or mixed coniferous and broadleaf plantings than in forests planted with all broadleaf species. The planting of coniferous species mainly affected the total relative abundance of abundant fungi, but that increasing the number of broadleaf species in planted mixed forests had less of an effect on the total relative abundance of abundant bacteria. The relative abundance of rare fungi was higher in the planted mixed forests than in the planted pure forests, and the relative abundance of rare fungi was higher in MPF than in MPPQ. Compared with planting single species, tree species mixing mainly increased the total relative abundance of rare fungi, and the changes in the total relative abundance of rare fungi were more sensitive to the changes in the number of broadleaf species in the planted mixed forests.

Analyses of soil microbial diversity showed that the α-diversity of abundant bacteria and rare fungi differed significantly between forest types, suggesting that forest types affect bacteria and fungi in the soil differently, and that soils of different forest types differ in their ability to enrich and utilize bacteria and fungi [42]. It has been shown that plants are able to enrich some microorganisms in the soil through specific root secretions and provide nutrients to microorganisms, thus driving the assembly of microbial communities in the soil [43]. The Shannon index of abundant bacteria in MPPQ was significantly lower than that in PFP and PFQ because the relative abundance of Proteobacteria in MPPQ was higher than that in PFP and PFQ. The total number of OTUs of abundant bacteria in the four forest types was basically the same, but the distribution of abundant bacterial species was not uniform in MPPQ. Similarly, the Shannon index of rare fungi in MPF was significantly higher than that in PFQ, and the relative abundance of Ascomycota in MPF was higher than that in PFQ, but the total number of OTUs of rare fungi in the four forest types was basically the same, and the distribution of rare bacterial species in MPF was not uniform. It has been shown that Ascomycota contains most known plant pathogens [44] and that the phylum can degrade persistent organic matter such as lignin [45]. Compared to PFQ, the tree species composition was richer in MPF, and more Ascomycota were needed in MPF to decompose lignin-containing litter in the soil. Whereas there was no significant difference in Shannon index among the four different forest types for abundant fungi, this suggests that Ascomycota associated with lignin degradation may occur in rare fungi and that future research on the function of Ascomycota in rare fungi is necessary. PCoA analyses showed a strong effect of forest type on soil microbial community β-diversity. Among the four forest types, there was no significant difference in the β-diversity of abundant bacteria and significant differences in the β-diversity of rare bacteria, abundant fungi, and rare fungi. The effect of changing forest types on bacterial β-diversity is reflected in rare bacteria, which have a wide variety of microorganisms in their communities, can metabolize a wider range of compounds [46,47], and are less susceptible to alteration by the external environment. For fungi, Ascomycota is effective in degrading organic residues in the soil [48] and breaking down waste into nutrients that can be absorbed. Although the relative abundance of Ascomycota in MPF-, MPPQ-, and PFQ-rich fungi was essentially the same and higher than that of PFP, Ascomycota in MPF rare fungi was significantly higher than that in the other three forest types. The rich composition of tree species in the canopy of MPF is also richer in soil litter than the other three forest types, thus requiring more Ascomycota to decompose the organic matter in the soil litter. This is consistent with the hypothesis that, even for microorganisms under the same phylum classification, soil microbial differences resulting from different canopy species in the forest are mainly in rare microorganisms.

### 4.2. Differences in the Assembly Processes of Abundant and Rare Microorganisms in Different Forest Types

There were differences in the assembly processes of abundant and rare microorganisms. The assembly of abundant bacterial and fungal communities in all four forest types was a stochastic process. This is because rare microbial communities have greater phylogenetic clustering compared to abundant microbial communities [11]. Environmental heterogeneity is a major factor influencing the assembly of soil microbial communities [49]. Abundant microbial communities have a higher relative abundance, are more extensive in their use of various nutrient resources in the soil [35,50], and are able to adapt to different environments. Abundant microbial communities with a wider ecological niche are less dependent on specific environmental conditions than rare microbial communities with a narrower ecological niche [28] and are better able to adapt to changes in the external environment [51]. The assembly of abundant microbial communities is more susceptible to stochastic processes [52,53], and their community assembly is driven by nutrient availability [42]; rare microbial community assembly processes are more influenced by interactions between organisms [54]. Rare bacteria and rare fungi are assembled by different processes. The assembly of rare bacterial communities in all four forest types is a deterministic process. This is due to the fact that bacteria grow and develop in a shorter period of time [55] and are able to spread rapidly [56]. Bacteria can adapt more quickly to environmental changes [57] and are more susceptible to environmental filtration or deterministic processes of biological interactions. However, it is important to note that the role of deterministic and stochastic processes in the assembly of soil microbial communities also depends on the particular microbial community and ecosystem [58]. In subsequent studies, the driving mechanism of the soil bacterial assembly process needs to be confirmed by adding investigations of deep forest ecosystems in other climatic zones. The assembly processes of rare fungal communities in the four forest types were influenced by both deterministic and stochastic processes, and the proportion of deterministic processes in rare fungal assembly was higher in planted mixed forests than in planted pure forests. This is due to the role of deterministic and stochastic processes within specific microbial communities and ecosystems [58]. Previous studies have shown that stochastic processes primarily drive fungal community assembly [59,60]. The assembly process of abundant fungal communities was stochastic in all four forest types of fungi. This suggests that stochastic processes mainly drive the assembly process of abundant fungal communities but not exclusively so for rare fungal communities. Abundant fungal communities are strongly regional in character and have a limited range of dispersal [61].

## 5. Conclusions

Forest soil microbial communities are the result of a combination of deterministic and stochastic ecological processes, and their abundant and rare microbial communities exhibit different ecological processes. Specifically, the assembly of abundant bacterial communities is mainly influenced by stochastic processes, the assembly of rare bacterial communities is mainly influenced by deterministic processes, the assembly of abundant fungal communities is mainly influenced by stochastic processes, and the assembly of rare fungal communities is influenced by a combination of deterministic and stochastic processes. Planted mixed forests increased the relative contribution of deterministic processes in the assembly of rare fungal communities compared to planted pure forests. This study reveals differences in the composition and diversity of abundant and rare microbial communities in different forest types and determines the relative contributions of deterministic and stochastic processes in the assembly of abundant and rare microbial communities between different forest types. These results contribute to the understanding of the complex roles of soil microorganisms in forests, can provide a deeper understanding of soil microbial communities in forest ecosystems, and can provide a theoretical basis for forest management in planted mixed forests. However, since this study was conducted only in the Zhongtiao Mountain region, research on the process of assembling forest abundant and rare soil microorganisms in other regions in the warm temperate continental climate zone is currently lacking. In future studies, the scope of investigations should be expanded, taking into account the effects of seasonal variations on the assembly processes of abundant and rare microorganisms in soils, and the study of soil nitrogen fixation, organic matter decomposition, and microorganism symbiosis in order to broaden the applicability of forest management practices.

## Data Availability

The original contributions presented in this study are included in the article/Supplementary Material. Further inquiries can be directed to the corresponding author.

## Supplementary Materials

The following supporting information can be downloaded at: https://www.mdpi.com/article/10.3390/microorganisms13081911/s1, Figure S1. Dilution curves for (a) bacteria and (b) fungi signature sequences in different forest types. Figure S2. β-dispersion test of soil microorganisms in different forest types. (a) abundant bacteria, (b) rare bacteria, (c) abundant fungi and (d) rare fungi. Table S1. Microbial sequencing information of four forest types. Table S2. Number of OTUs for total, abundant and rare bacteria and fungi. Table S3. Shannon index of abundant bacteria, rare bacteria, abundant fungi and rare fungi. Table S4. NTI of abundant bacteria, rare bacteria, abundant fungi and rare fungi.
