# Supplementary material for: Assembly of Abundant and Rare Bacterial and Fungal Communities in Different Typical Forest Types in the Zhongtiao Mountains
Source: Microorganisms. 2025 Aug 16;13(8):1911. doi: 10.3390/microorganisms13081911 (PMC12388160; doi:10.3390/microorganisms13081911)
Supplement: Supplementary file 1 [file microorganisms-13-01911-s001.zip › microorganisms-3782407-supplementary.pdf]

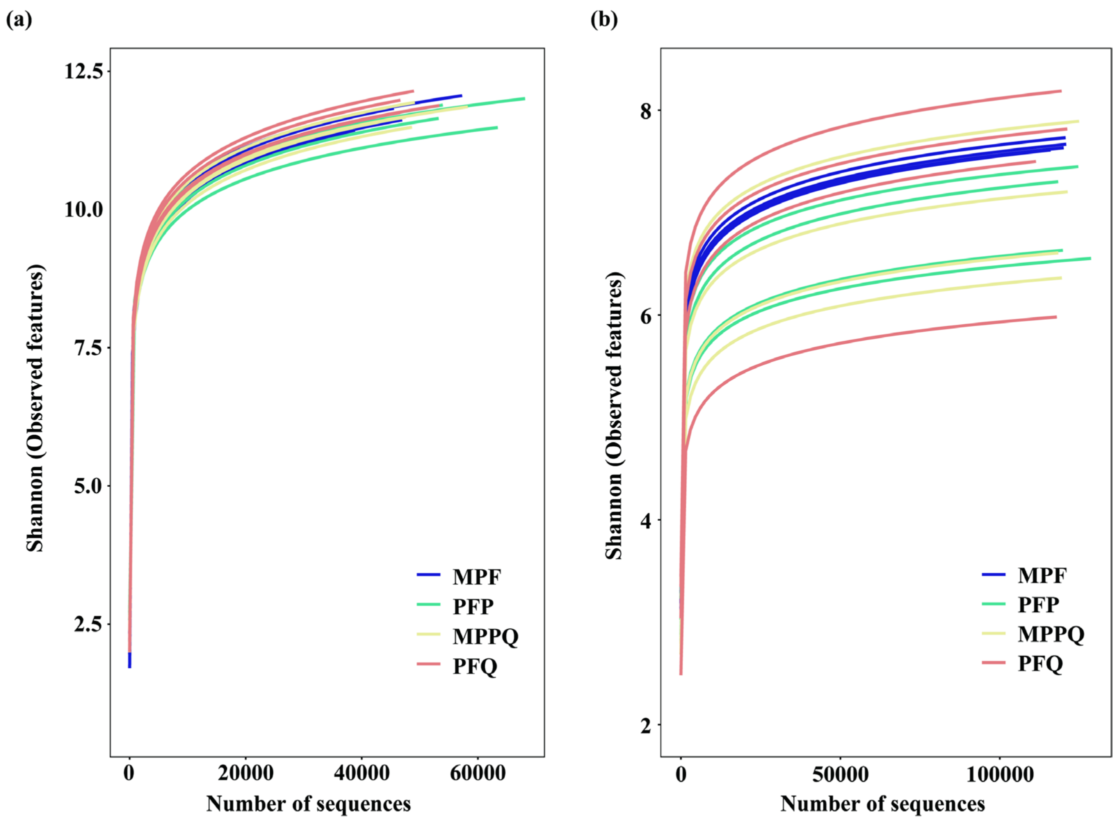

Figure S1. Dilution curves for (a) bacteria and (b) fungi signature sequences in different forest types.

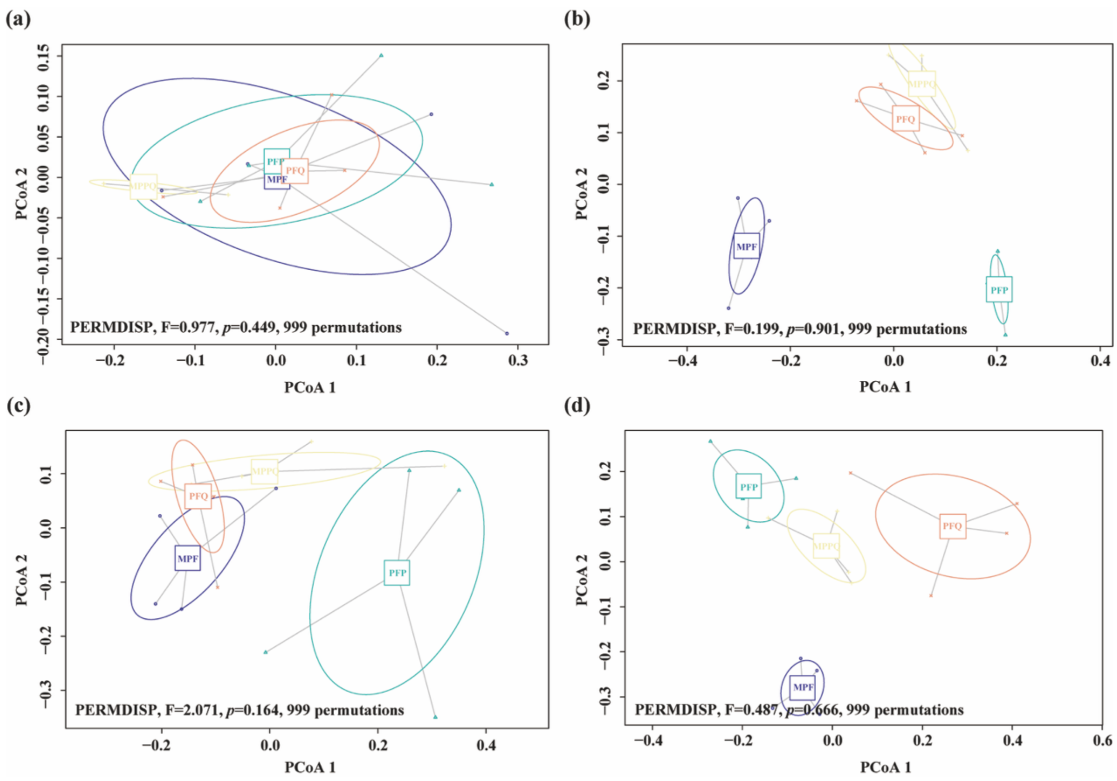

Figure S2. β-dispersion test of soil microorganisms in different forest types. (a) abundant bacteria, (b) rare bacteria, (c) abundant fungi and (d) rare fungi.

Table S1. Microbial sequencing information of four forest types.

	MPF	PFP	MPPQ	PFQ
Bacteria reads	88499±15906	106852±1847	94093±2684	92822±12201
Bacteria Good's coverage	99.67%	99.69%	99.61%	99.54%
Fungi reads	127561±2159	131573±4773	128278±2297	124600±4192
Fungi Good's coverage	99.99%	99.99%	100%	100%

Table S2. Number of OTUs for total, abundant and rare bacteria and fungi.

	OTUs taxa	MPF	PFP	MPPQ	PFQ
Bacteria	Total	48337	60976	51990	50187
	Abundant	516	749	688	581
	Rare	5673	8224	6049	6693
Fungi	Total	119336	122802	120964	117419
	Abundant	15338	10171	14823	15951
	Rare	1461	1014	1101	907

Table S3. Shannon index of abundant bacteria, rare bacteria, abundant fungi and rare fungi.

OTUs taxa	MPF	PFP	MPPQ	PFQ	*p*-value
Abundant bacteria	0.98±0.09ab	1.02±0.05a	0.85±0.07b	1.01±0.06a	0.019*
Rare bacteria	7.52±0.38a	7.81±0.09a	7.61±0.09a	7.71±0.22a	0.346
Abundant fungi	1.53±0.15a	1.62±0.13a	1.38±0.08a	1.49±0.17a	0.130
Rare fungi	5.40±0.17a	5.07±0.30ab	5.13±0.13ab	4.96±0.16b	0.049*

Note: Different letters indicate that shannon index was significantly different ($p < 0.05$) among the four forest types. Asterisks denote significance levels (*$p < 0.05$).

Table S4. NTI of abundant bacteria, rare bacteria, abundant fungi and rare fungi.

OTUs taxa	MPF	PFP	MPPQ	PFQ	*p*-value
Abundant bacteria	0.17±0.85a	-0.11±1.23a	1.05±0.10a	0.82±0.62a	0.204
Rare bacteria	11.47±1.16a	9.16±0.48b	9.33±0.86b	8.92±0.75b	0.004**
Abundant fungi	1.01±0.11a	0.76±0.44a	0.47±0.28a	0.69±0.53a	0.286
Rare fungi	1.57±1.02a	0.01±1.59a	-1.59±2.59a	-1.71±1.25a	0.060

Note: Different letters indicate that NTI was significantly different ($p < 0.05$) among the four forest types. Asterisks denote significance levels (**$p < 0.01$).
